# The Added Value of [^18^F]FDG PET/CT in the Management of Invasive Fungal Infections

**DOI:** 10.3390/diagnostics11010137

**Published:** 2021-01-17

**Authors:** Alfred O. Ankrah, Dina Creemers-Schild, Bart de Keizer, Hans C. Klein, Rudi A. J. O. Dierckx, Thomas C. Kwee, Lambert F. R. Span, Pim A. de Jong, Mike M. Sathekge, Andor W. J. M. Glaudemans

**Affiliations:** 1 Medical Imaging Center, University of Groningen, University Medical Center Groningen, 9700 AC Groningen, The Netherlands; hanscklein@gmail.com (H.C.K.); r.a.dierckx@umcg.nl (R.A.J.O.D.); t.c.kwee@umcg.nl (T.C.K.); a.w.j.m.glaudemans@umcg.nl (A.W.J.M.G.); 2 Department of Nuclear Medicine, University of Pretoria, Steve Biko Academic Hospital, Pretoria 0001, South Africa; mike.sathekge@up.ac.za; 3 National Centre for Radiotherapy Oncology and Nuclear Medicine, Korle Bu Teaching Hospital, Accra GA-222 7974, Ghana; 4 Department of Internal Medicine, Groene Harte Hospital, 2803 HH Gouda, The Netherlands; dinaschild@hotmail.com; 5 Department of Radiology and Nuclear Medicine, Utrecht University, University Medical Center Utrecht, 3508 GA Utrecht, The Netherlands; B.dekeizer@umcutrecht.nl (B.d.K.); p.dejong@umcutrecht.nl (P.A.d.J.); 6 Department of Internal Medicine, Division of Hematology, University of Groningen, University Medical Center Groningen, 9700 AC Groningen, The Netherlands; l.f.r.span@umcg.nl

**Keywords:** invasive fungal infections, PET, CT, MRI, ultrasound, chest X-ray

## Abstract

Anatomy-based imaging methods are the usual imaging methods used in assessing invasive fungal infections (IFIs). [^18^F]FDG PET/CT has also been used in the evaluation of IFIs. We assessed the added value of [^18^F]FDG PET/CT when added to the most frequently used anatomy-based studies in the evaluation of IFIs. The study was conducted in two University Medical Centers in the Netherlands. Reports of [^18^F]FDG PET/CT and anatomy-based imaging performed within two weeks of the [^18^F]FDG PET/CT scan were retrieved, and the presence and sites of IFI lesions were documented for each procedure. We included 155 [^18^F]FDG PET/CT scans performed in 73 patients. A total of 216 anatomy-based studies including 80 chest X-rays, 89 computed tomography studies, 14 magnetic resonance imaging studies, and 33 ultrasound imaging studies were studied. The anatomy-based studies were concordant with the [^18^F]FDG PET/CT for 94.4% of the scans performed. [^18^F]FDG PET/CT detected IFI lesions outside of the areas imaged by the anatomy-based studies in 48.6% of the scans. In 74% of the patients, [^18^F]FDG PET/CT added value in the management of the IFIs.

## 1. Introduction

Invasive fungal infections (IFIs) cause significant morbidity and mortality, especially in immunocompromised hosts [1]. IFIs are caused by different organisms that can be generally classified into the yeasts and molds. The most common yeast causing IFIs is *Candida albicans*, although the prevalence of other *Candida species* is increasing. In the case of the molds, *Aspergillus fumigatus* is the most common species, but other Aspergillus species and non-Aspergillus molds are becoming more prevalent. The population of patients with IFIs is increasing because there has been an increase in the population at risk of these predominantly opportunistic infections. The population at risk of IFIs includes patients with primary immunodeficiency disorders and patients with immune deficiencies due to hematologic malignancies, chemotherapy, hematologic and solid organ transplantation, steroids and other immunosuppressive therapy or diabetes mellitus. Fungi usually invade the body by the respiratory route; however, fungi may also translocate from the skin or mucosa to sterile sites in the body as a consequence of invasive procedures such as central venous catheter insertion or trauma. IFIs may result in severe infections, especially in hematologic patients, that are difficult to diagnose and treat. They may cause severe morbidity and even be fatal if not treated adequately. For some specific underlying conditions, mortality rates exceeding 70% have been reported for IFIs [2,3].

The lung is the most common site of IFIs, but IFIs can affect any part of the body. Imaging plays an essential role in the management of IFIs [4]. Fluorine-18 fluorodeoxyglucose positron emission tomography integrated with computed tomography ([^18^F]FDG PET/CT) has been used in the management of IFIs [5,6]. [^18^F]FDG PET/CT, as a whole-body hybrid imaging technique, allows functional data from positron emission tomography (PET) to be correlated with anatomic data from computed tomography (CT) [7]. [^18^F]FDG PET/CT has the advantage of being able to scan most body regions in one sitting in addition to providing metabolic information. On the other hand, most anatomy-based imaging modalities often image fewer regions of the body per imaging session in routine clinical practice. Anatomy-based imaging procedures are more commonly used in imaging IFIs during the management of the infections. These modalities include chest X-rays, high-resolution computed tomography of the chest (HR CT), CT of the chest and other parts of the body, magnetic resonance imaging (MRI), and ultrasound imaging (US) [4,8,9,10]. Recent data suggest that [^18^F]FDG PET/CT may add extra information to the data supplied by anatomy-based studies to help the management of IFIs [2,5,6,11,12,13,14]. There are relatively few large studies available that have examined the use of [^18^F]FDG PET/CT in IFIs [6,11,12,13,14]. All the studies suggest that [^18^F]FDG PET/CT may be useful in staging and monitoring IFIs. Furthermore, one study evaluated the sensitivity, specificity and predictive value of [^18^F]FDG PET/CT in the diagnosis of IFIs [6]. Another study compared [^18^F]FDG PET/CT to conventional CT [12], and our group previously evaluated metabolic PET parameters in the monitoring of IFIs [11].

In this study, an expansion of our previous study, we evaluated the added value of [^18^F]FDG PET/CT used along with anatomy-based imaging modalities that are commonly used in the routine clinical management of IFIs.

## 2. Materials and Methods

The scans of patients diagnosed with IFIs who also underwent [^18^F]FDG PET/CT as part of the management of the IFIs were retrospectively reviewed. We included patients who had proven, probable or possible IFIs according to the revised European Organization for Research and Treatment of Cancer/Invasive Fungal Infections Cooperative Group and the National Institute of Allergy and Infectious Diseases Mycoses Study Group (EORTC/MSG) classification [15]. The study was conducted in two Medical Centers in the Netherlands, The University Medical Center Groningen (UMCG) and The University Medical Center Utrecht (UMCU). All chest X-rays, HR CT, CT scans, MRI, and ultrasound imaging that were performed within two weeks of the [^18^F]FDG PET/CT were retrieved, and the results were documented. The electronic patient files of all the included patients were retrieved and scanned for relevant clinical data. Board-certified nuclear medicine physicians and radiologists reported the images as part of routine clinical practice. The images used in the study were acquired between October 2009 and March 2018. Given the retrospective nature and use of routine care data, no formal ethical approval or informed consent was required (UMCG 201600073). The data were handled according to general data protection regulation (GDPR) requirements.

All [^18^F]FDG PET/CT scans were acquired according to the EANM (European Association of Nuclear Medicine) guidelines [16], all on hybrid PET/CT camera systems (Siemens Biograph, Siemens Heathineers). The CT component of the [^18^F]FDG PET/CT was non-contrast enhanced. The anatomy-based imaging was conducted according to the departmental protocols. The [^18^F]FDG PET/CT study was considered to be concordant with the anatomy-based study if abnormal lesions due to IFIs were detected in both studies or if no lesion could be attributed to an IFI on both. We also recorded [^18^F]FDG PET/CT studies in which abnormal IFI lesions were present at sites that were not imaged by the other imaging studies performed within two weeks of the PET/CT.

We defined added value when the metabolic information provided by the study allowed the follow-up of the activity of IFI lesions or allowed clinical decisions on patient management to be made in either stopping, prolonging or changing antifungal treatment in patients with IFIs. Potentially, [^18^F]FDG PET/CT was considered to add value to a given anatomy-based imaging modality when it detected IFI lesions outside the region imaged by the anatomy-based study.

The final diagnosis of the patient was based on the revised definitions of the EORTC/MSG classification for invasive fungal infections [15]. Patients who were positive for respiratory specimen fungal cultures or positive for fungal cultures from biopsies from sterile sites were classified as having proven IFIs. Patients with clinical factors putting them at risk of developing IFIs who were positive for blood or respiratory galactomannan and/or showed serological evidence of IFIs and suggestive imaging findings were classified as probable IFI patients. Patients with clinical factors putting them at risk of developing IFIs and imaging findings in support of IFIs but who were not positive for respiratory or blood galactomannan and did not show serological evidence to support the diagnosis were defined as having possible IFIs. The patients with the possible and probable criteria must have also demonstrated responses to antifungal treatment on follow-up according to either clinical features or imaging.

We use descriptive statistics to present the data. The number of patients, [^18^F]FDG PET/CT scans, anatomy-based imaging modalities, underlying predisposing conditions, indications for the study and concordance between [^18^F]FDG PET/CT and anatomy-based studies are expressed as percentages or frequencies, and the data are presented in tables.

## 3. Results

### 3.1. Patient Demography and IFI Characteristics

**Figure 1:**
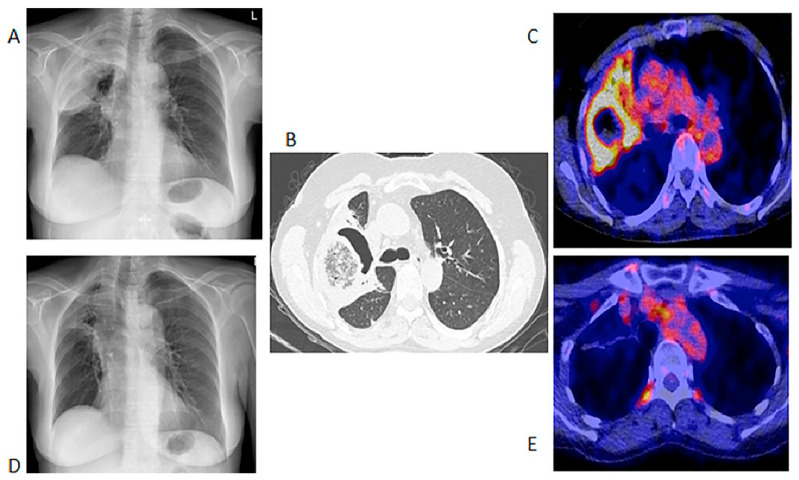
Chest X-ray, HR CT and [^18^F]FDG PET/CT of a 60-year-old female with acute myeloid leukemia on chemotherapy. She had a proven fungal infection (*Alternaria alternata*). (**A**)—Initial chest X-ray. (**B**)—Initial HR CT scan performed before the [^18^F]FDG PET/CT. (**C**)—Fused axial image of [^18^F]FDG PET/CT of the chest. After 2 months of antifungal therapy. (**D**)—Chest X-ray showing response with a residual lesion. (**E**)—Fused axial [^18^F]FDG PET/CT showing an almost complete metabolic response in the IFI lesion. The data provided by [^18^F]FDG PET/CT at follow-up were deemed adequate, so patient did not require a repeat HR CT.

We included 155 [^18^F]FDG PET/CT studies from 73 patients who were diagnosed with IFIs. The majority of the patients (*n* = 49, 67%) were males, and the median age at the time of the scan was 56 years, with a range from 9 months to 76 years. Fifty-nine (81%) patients were from UMCG, and fourteen (19%) were from UMCU. Forty-one patients had a single [^18^F]FDG PET/CT in the assessment of IFIs. The IFIs were classified according to the revised EORTC/MSG criteria, and the results are displayed in Table 1. Thirty-two patients had repeated [^18^F]FDG PET/CT studies performed to monitor the responses (Table 2). Fourteen (45.1%) of the patients who had repeated [^18^F]FDG-PET to monitor the treatment of the IFIs did not have proven IFIs as per the EORTC/MSG criteria. The conditions underlying the IFIs are tabulated in Table 1. There were five major indications for [^18^F]FDG PET/CT study. These five indications are displayed in Table 2 below. Figure 1 shows the initial chest X-ray, HR CT and fused axial [^18^F] FDG PET/CT through the chest of a patient with a proven fungal infection and the follow up [^18^F]FDG PET/CT study.

### 3.2. [^18^F]FDG PET/CT Studies

**Figure 2:**
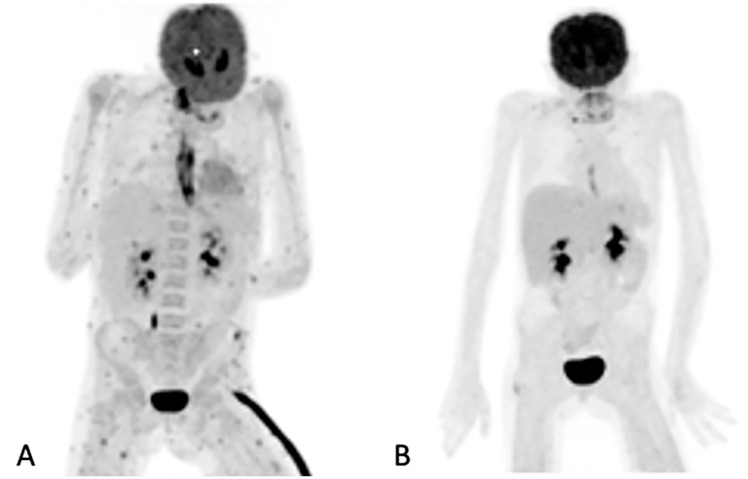
Maximum intensity projection (MIP) of [^18^F]FDG images of a 10-year-old girl on chemotherapy for acute lymphocytic leukemia. She had a persistent fever unresponsive to antibiotics. HR CT not shown (was unremarkable). The initial study (**A**) revealed widespread, multiple, small, metabolically active foci in the muscles and esophagitis, which were later found to be due to *Candida dubliniesis* upon biopsy. The lesions were not detected by the corresponding CT of the [^18^F]FDG PET/CT study. A follow-up [^18^F]FDGPET/CT study (**B**) after 6 weeks of antifungal treatment showed a very good metabolic response, with a single residual focus in the right gluteus and the resolution of the esophagitis. No anatomy-based study was performed together with the follow-up [^18^F]FDG PET/CT study (**B**).

Of the 155 [^18^F]FDG PET/CT studies that were analyzed, 34 (22%) had no anatomy-based imaging (Table 3). Of the 34 [^18^F]FDG PET/CT studies that had no anatomy-based imaging, 28 (82.4%), were scans that were performed for the follow-up of IFI lesions that had been previously noted by previous imaging. The remaining four were two in patients being prepared for ASCT, and two in two patients for which [^18^F]FDG PET/CT had found metabolic activity in residual IFI lesions. Figure 2 shows a patient for which [^18^F]FDG PET/CT only was used to follow up because the IFI lesions could only be detected by the metabolic component of the study. Table 3 outlines the number of anatomy-based studies that were performed for each [^18^F]FDG PET/CT scan studied and the concordance between the [^18^F]FDG PET/CT and anatomy-based study at the site of the study.

### 3.3. Anatomy-Based Studies

A total of 216 anatomy-based imaging studies were performed within two weeks of the [^18^F]FDG PET/CT studies. Imaging of the chest was performed in 66% (142/216) of these studies by chest X-rays (*n* = 80), chest CT and HR CT (*n* = 62). Abdominal imaging was performed in 24% (51/216) of the studies by ultrasound imaging (*n* = 30) and abdominal CT (*n* = 21). Brain imaging was performed in 5% (10/216) by MRI. Evaluation of the facial sinuses, intervertebral discs and heart valves was performed in 4% (9/216), by CT scans (3/216), MRI (3/216) and ultrasound of the heart (echocardiograph) (3/216). MRI was used to evaluate the soft tissue of the upper limb by MRI in one patient who had an IFI of *Pleurostomorpha richardsiae* following a puncture wound. Table 4 shows the different forms of anatomy-based imaging evaluated, the numbers of [^18^F]FDG PET/CT tests performed within two weeks, the numbers of anatomy-based studies that were concordant with the [^18^F]FDG PET/CT at the sites imaged by the anatomy-based studies, and the numbers of anatomy-based studies for which [^18^F]FDG PET/CT detected lesions outside the sites imaged by the anatomy-based studies.

### 3.4. Discordance between [^18^F]FDG PET/CT and Anatomy-Based Imaging

[^18^F]FDG PET/CT detected abnormal pulmonary lesions due to IFIs in 7.5% (6/80) of the chest X-rays performed that were not detected in the corresponding X-rays. Nine percent (3/33) of the US-based studies were not concordant with the [^18^F]FDG PET/CT, with lesions present on [^18^F]FDG PET/CT but not on US. [^18^F]FDG PET/CT did not detect IFI lesions for 21% (3/14) of the intracerebral IFIs that were detected by diffusion-weighted MRI.

### 3.5. Added Value Due to the Metabolic Assessment of Lesions by Patient

**Figure 3:**
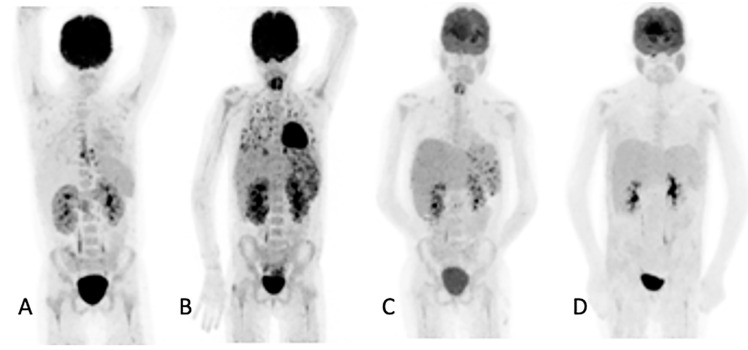
Ten-year-old female with acute myeloid lymphoma who developed a fever, on chemotherapy, with no response to antibiotics. An abdominal ultrasound demonstrated bilaterally enlarged kidneys. Initial [^18^F]FDG PET/CT MIP image (**A**) showed focal metabolic uptake in kidneys, spleen and esophagus. She was started on antifungal treatment, but the fever persisted, and a repeat study (**B**) showed the worsening of renal splenic lesions with no pulmonary lesions. The antifungal treatment was changed; the fever subsided, and the serum infective markers decreased. A repeat [^18^F]FDG PET/CT performed after 6 weeks (**C**) showed resolution of the pulmonary and most of the renal and splenic lesions. The treatment was continued for another month, and a follow-up [^18^F]FDG PET/CT (**D**) showed a complete metabolic response that helped the clinician to end the antifungal treatment.

**Figure 4:**
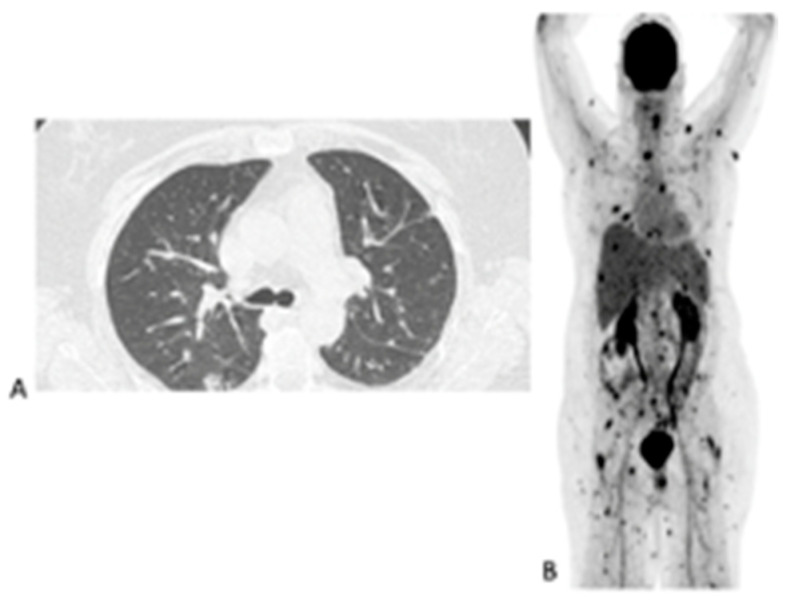
Sixty-three-year-old female with acute myeloid leukemia on treatment for pulmonary aspergillosis. [^18^F]FDG PET/CT was performed because there was unexplained fever, and the IFI lesions seen on HR CT (**A**) were similar to in the HR CT scan performed a month and 2 months earlier. [^18^F]FDG PET/CT scan (**B**) showed multiple IFI lesions outside the thorax, providing an explanation for the persistent fever.

In the 32 patients that had repeated [^18^F]FDG PET/CT imaging to assess the responses, the follow-up helped the clinicians to decide to stop, continue or change antifungal therapy (Figure 3). [^18^F]FDG PET/CT detected previously unknown IFI lesions in five patients of the 18 that were referred for the staging of the infection, which led to therapy prolongation in four and a change in therapy in one. In the 10 patients with indications of unexplained fevers or increasing infective markers, [^18^F]FDG PET/CT was found to be useful in four patients, as it guided the biopsy that led to the diagnosis of the IFI (see Figure 4). In the seven patients that were being prepared for ASCT, [^18^F]FDG PET/CT was considered to add value for all. In four patients, the [^18^F]FDG PET/CT allowed clinicians to proceed with the procedure; in two others, further antifungal therapy was provided with follow-up with [^18^F]FDG PET/CT, and a biopsy was conducted in one of the patients before ASCT was performed. Finally, in the six patients where scans was performed to assess metabolic activity in residual anatomic lesions, [^18^F]FDG PET/CT was considered useful. There was a complete metabolic response in five patients, and in two patients, further antifungal treatment was instituted. Table 5 tabulates the patients for which [^18^F]FDG PET/CT was deemed to have added value. Figure 4 shows a female patient with acute myeloid leukemia while on antifungal therapy, where [^18^F]FDG PET/CT explained the increasing infective markers by demonstrating multiple IFI lesions outside the thorax.

## 4. Discussion

There are currently many imaging procedures the clinician has available for imaging IFIs. In this current study, we demonstrated that [^18^F]FDG PET/CT adds value to anatomy-based studies. We also showed that [^18^F]FDG PET/CT was frequently concordant with other imaging study modalities that are commonly used in the assessment of pathology due to IFIs, but also detected lesions outside the anatomy-based scan regions in almost half of the patients. Furthermore, considering all the patients together, [^18^F]FDG-PET/CT was found to add value in the management of 74% of the IFI patients.

The diagnosis of fungal infections is challenging. The direct isolation of fungi from sterile sites is not always possible. Antifungal therapy is often initiated in patients without proven fungal infections. This strategy helps to reduce the morbidity and mortality associated with delays in initiating treatment for antifungal infections. However, this may lead to the unnecessary administration of antifungal treatment, which may be associated with adverse effects and the development of resistance. Pre-emptive therapy that is not diagnosis driven is often used. The EORTC/MSG has a revised classification for the diagnosis of IFIs [15]. The identification and monitoring of the antifungal treatment of IFI lesions in the probable and possible groups would be of great value to clinicians. [^18^F]FDG PET/CT would identify IFI lesions and assess disease activity over time. This would help to monitor responses to antifungal therapy and identify early on if the therapy needed to be changed and may even identify alternative pathologies by directing the biopsies of unresponsive lesions [5,11]. In our study, 45% of the patients had either possible or probable IFIs as per the EORTC/MSG criteria, and had repeated [^18^F]FDG PET/CT scans to monitor therapy.

Our study found that in over 20% of the [^18^F]FDG PET/CT scans performed, there was no other imaging modality performed within two weeks. This may suggest that the information derived from the [^18^F]FDG PET/CT was sufficient for clinical decisions to be made. Over 80% of these [^18^F]FDG PET/CT scans were performed as follow-up studies once abnormal IFI lesions had been previously identified by any imaging modality. This emphasizes not only the importance of [^18^F]FDG PET/CT as an important tool in following up disease, but also the ability of [^18^F]FDG PET/CT to replace most other imaging modalities as the imaging modality of choice in the follow-up of IFIs during antifungal treatment.

[^18^F]FDG PET/CT, as a whole-body imaging procedure, enables the detection of IFIs at different sites of the body in a single imaging session. The metabolic uptake from the PET study makes it very useful for monitoring disease activity over time [17]. By contrast, anatomy-based imaging procedures are usually limited to one region per study. CT and MRI scans of the whole body are possible; however, this is not routine clinical practice, and this study was performed to determine the value of adding [^18^F]FDG PET/CT to the anatomy-based imaging used in the routine clinical setting [18]. Our study found that [^18^F]FDG PET/CT detected lesions outside the regions imaged by the anatomy-based studies in almost 50% of the studies (Table 4).

The plain chest radiograph is readily available and, often, one of the earliest tests requested, as IFIs commonly affect the lungs. The plain chest radiograph, however, is less useful compared to other procedures such as CT of the chest in the early stages of IFIs [19,20]. In our study, [^18^F]FDG PET/CT detected IFI lesions in 7.5% of the chest X-rays reported as normal.

CT of the chest, especially HR CT, has been used in the management of IFIs and has been incorporated in international guidelines for the management of IFIs [9,20,21]. The lung is the most common site of IFIs. Some authors have recommended the combination of HR CT and FDG PET/CT when evaluating pulmonary lesions to increase sensitivity and specificity [22]. A previous report demonstrated [^18^F]FDG uptake in IFI lesions that were not seen on the corresponding CT [23]. In our study, we demonstrated 100% concordance of the CT, both thoracic and extra thoracic CT, with [^18^F]FDG PET/CT. [^18^F]FDG PET/CT has CT information as part of the data it acquires. The anatomic data from CT are combined with functional data from PET, providing the advantages of both imaging modalities in a single examination. The CT component in our study was a non-contrast-enhanced diagnostic CT, and combined with [^18^F]FDG PET, it was able to detect all lesions due to IFIs through the CT studies conducted without PET. In addition, our study found that [^18^F]FDG PET/CT detected lesions outside the field of view of CT in 36% and 52% of the thoracic and extra thoracic CT, representing 40% (36/89) of the total number of CT scans performed, potentially adding value to the CT studies. Thus, acquiring only [^18^F]FDG-PET combined with the non-contrast-enhanced CT of the whole body is, in our opinion, enough.

MRI is another crucial imaging modality for the evaluation of IFIs. In the brain and sinuses, where physiological [^18^F]FDG uptake may compromise the detection of IFI foci by PET/CT, MRI is beneficial. The excellent soft-tissue resolution of MRI allows the better visualization of small, thin structures such as the meninges, and the different acquisition sequences of MRI are useful in the early diagnosis of intracerebral IFI [2,4], as our study demonstrated. The advantages of each imaging procedure must be carefully considered when imaging a patient with known or suspected IFIs, taking into account the limitations of each imaging study. In our study, diffusion-weighted MRI detected abnormal brain and meningeal IFI lesions in over 20% of the MRI scans performed, which were not detected by [^18^F]FDG PET/CT. The uptake of [^18^F]FDG in the brain precluded the detection of these lesions, which could still not be seen when the [^18^F]FDG PET/CT was reviewed with the results of the MRI known. However, [^18^F]FDG PET/CT detected IFI lesions outside the regions of interest imaged in over 70% of the MRI scans in our study (Table 5). [^18^F]FDG PET/CT may add value to MRI imaging by detecting sites of disease outside the regions evaluated by the MRI that may potentially affect management.

The US of the abdomen is also used in imaging IFIs located in the liver, spleen or kidneys in IFIs such as chronic disseminated candidiasis. US imaging can be easily and quickly performed at the bedside. However, it lacks sensitivity in patients with neutropenia and is operator dependent [8,24]. [^18^F]FDG PET/CT, as shown in our study, was useful for the detection of IFIs both at the site and beyond the region of interest of the US study (Table 5). However, 9.1% of the US performed was unable to visualize IFI lesions demonstrated by the [^18^F]FDG PET/CT. The IFIs in those cases may have been in early stages; previous studies have reported CT, a component of [^18^F]FDG PET/CT, detecting more cases of IFI compared to US [8,25].

Occult lesions in IFIs could be potentially devastating if intense immunosuppressive procedures such as ASCT are considered as part of their management [26]. In our study, [^18^F]FDG PET/CT served as a gate keeper in all the patients for whom ASCT was being considered. Both positive and negative IFI findings in the [^18^F]FDG PET/CT helped in clinical decision making and added value to the management of IFIs. [^18^F]FDG PET/CT could be used to provide clinical direction when the intervention being undertaken could cause the dissemination of the IFI.

Anatomic lesions may persist in patients with IFIs despite the resolution of the infection; this may lead to undue prolongation in antifungal therapy. As metabolic changes precede anatomic changes, [^18^F]FDG PET/CT can provide information to enable clinical decisions on antifungal treatment to be made as quickly as possible [5,27]. These changes will allow clinicians to switch treatments when therapy is ineffective and stop treatments early to avoid adverse effects and reduce the durations and costs of antifungal therapy. In our study, we showed that [^18^F]FDG PET/CT was useful in assessing metabolic activity in all the patients referred for the assessment of IFI lesions.

The findings of our study compare favorably with other studies in the literature. Douglas et al. compared [^18^F]FDG PET/CT to conventional CT in detecting IFIs and guiding the management of the infections [12]. In their study, [^18^F]FDG PET/CT localized occult disease and detected IFI dissemination to other organs in 40% and 38% of patients, respectively [12]. This indirectly compares with our findings, where we detected IFI lesions outside the regions imaged by the CT scans in 40% of the [^18^F]FDG PET/CT performed within two weeks of the CT. The study by Douglas et al. also found that [^18^F]FDG PET/CT detected significantly more sites of IFI dissemination compared to CT (35% vs. 5%, *p* < 0.001). The authors concluded that [^18^F]FDG PET/CT was useful in both the detection of occult disease and evaluation of IFI dissemination [12]. In another study, [^18^F]FDG PET/CT was found to influence the diagnostic work for IFIs in 55% of patients, led to a change in antifungal therapy in 15% of patients and led to a cessation of antifungal therapy in 31% of patients [6]. The findings in this study are similar to those in our study, where [^18^F]FDG PET/CT added value in 74% of patients. Our group previously reported the role of [^18^F]FDG PET/CT in monitoring responses in patients with IFIs [11]. In that study, we evaluated 28 patients, part of this current study, who had repeated studies, including one at baseline and at least one at another time-point during treatment. We found that [^18^F]FDG was useful in monitoring therapy in IFIs, resulting in a change in treatment in almost 50% of the patients. We reported how the global total lesion glycolysis may have prognostic value in antifungal therapy [11].

This study has some limitations. The first limitation is the retrospective nature of the study. The clinical decisions made about IFI management were based not on [^18^F]FDG PET/CT or other imaging scans alone but on a combination of clinical data. The contribution of the [^18^F]FDG PET/CT to the decision making is difficult to assess in this scenario. The relatively small numbers affect the generalizability of these results. However, IFIs are relatively rare, and these results form the basis on which prospective multi-centered studies can be recommended to determine the full potential of [^18^F]FDG PET/CT in invasive fungal infections.

Additionally, the lack of histological confirmation in all patients with IFIs is a limitation. However, it is not always possible to obtain full proof by biopsies in all the lesions present in clinical practice.

## 5. Conclusions

[^18^F]FDG PET/CT added value to the management of IFIs in 74% of patients; it detected lesions outside the regions imaged by the anatomy-based studies in almost half of the studies.

## Figures and Tables

**Table 1:** Types, classifications and underlying predisposing factors for invasive fungal infections (IFIs).

	Number (%) (*n* = 73)
**Type of IFI**	
Aspergillosis	48 (66%)
Candidiasis	21 (29%)
Other	4 (5%)
*Crytococcus neoformans* *Hormografiella aspergillata* *Pleurostomorpha richardsiae* *Alternaria alternata*	1
	1
	1
	1
**Classification of IFI**	
Patients with proven IFIs	42 (58%)
Patients with probable IFIs	19 (26%)
Patients with possible IFIs	12 (16%)
**Underlying predisposing factor**	
Acute leukemia	27 (37%)
Hematologic conditions excluding acute leukemia	18 (25%)
Solid-organ transplantation	10 (14%)
Invasive procedures	4 (4%)
High-dose steroids	4 (5%)
Intense chemotherapy	3 (4%)
Lung cavitation	2 (3%)
No clear underlying conditions	5 (7%)

**Table 2:** Indication for the [^18^F]FDG PET/CT study.

Indication	Number (%) (*n* = 73)
Monitor response to antifungal therapy	32 (44%)
Stage infection	18 (25%)
Unexplained fever or increasing infective markers	10 (14%)
Evaluation for ASCT (Allogeneic stem cell transplantation)	7 (10%)
Detect active disease in residual lesions	6 (8%)

**Table 3:** Number of anatomy-based studies per [^18^F]FDG PET/CT.

Anatomy-Based Studies for Each [^18^F]FDG PET/CT Scan Performed	[^18^F]FDG PET/CT Scans Performed	[^18^F]FDG PET/CT Scans That Were Concordant with All the Anatomy-Based Studies Performed (%)
0	34 (21.9%)	*n*/A
1	43 (27.7%)	42 (97.6%)
2	62 (40.0%)	53 (85.5%)
3	15 (9.7%)	13 (86.6%)
4	1 (0.1%)	1 (100%)
Total	155 (100%)	109 of 121 (90.1%)

**Table 4:** Showing numbers of [^18^F]FDG PET/CT studies performed for each anatomy-based study, concordance, and numbers of [^18^F]FDG PET/CT tests that detected IFI lesions outside the regions of interest of the anatomy-based studies.

Anatomy-Based Study	[^18^F]FDG PET/CT Performed ^1^ (*n*, %)	Concordance with [^18^F]FDG PET/CT (*n*, %)	[^18^F]FDG PET/CT That Detected Lesions outside Region Imaged ^2^ (*n*, %)
Chest X-ray	80 (37%)	74 (92.5%)	37 (42.3%)
HR CT and thoracic CT	62 (29%)	62 (100%)	22 (35.5%)
Extra thoracic CT scan	27 (13%)	27 (100%)	14 (51.8%)
MRI	14 (6%)	11 (78.6%)	10 (71.4%)
Ultrasound imaging	33 (15%)	30 (90.9%)	22 (66.7%)
Overall	216 (100%)	204 (94.4%)	105 (48.6%)

^1^ Percentage of number of specific anatomy-based studies out of all anatomy-based studies performed within two weeks of [^18^F]FDG PET/CT. ^2^ Percentage of [^18^F]FDG PET/CT tests that detected lesions outside the site imaged by anatomy-based study.

**Table 5:** Patients for which [^18^F]FDG PET/CT was considered to add value.

Value Added	Number (%)
(Total number of patients)	73 (100%)
Assessing response to therapy	32 (44%)
Detected previously undiagnosed sites of IFIs	5 (7%)
Unexplained fever leading to biopsy-guided diagnosis of IFI	4 (5%)
Evaluation for ASCT	7 (10%)
Metabolic activity of residual IFI lesions on anatomy-based imaging	6 (8%)
Total patient added value for [^18^F]FDG PET/CT	54 (74%)

## Data Availability

De-identified patient data are available on request from the author.
